# “You teach us to listen,… but you don’t teach us about suffering”: self-care and resilience strategies in medical school curricula

**DOI:** 10.1007/s40037-014-0145-9

**Published:** 2014-11-14

**Authors:** Sue Outram, Brian Kelly

**Affiliations:** School of Medicine and Public Health, University of Newcastle, Callaghan, NSW 2308 Australia

**Keywords:** Medical curricula, Stress, Resilience, Self-care

## Abstract

This article examines the pre-vocational preparation of doctors to cope with the demands of clinical practice, drawing on literature from across a number of domains: mental health, psychological stress among medical students and medical practitioners; and self-care strategies in medicine curricula. High rates of psychological distress in medical students and medical practitioners were consistently reported. A number of questions remain pertinent to medical education: how does the experience of medical education impact on this level of distress, and possibly exacerbate pre-existing student vulnerabilities? What will help future doctors respond to, and cope with, suffering in their patients? Can the formal curriculum build resilience? Medical schools and educators have a responsibility to address these questions and to provide effective self-care curricula. In this review promising interventions such as mindfulness training are reported, frameworks to guide self-awareness in medical students are suggested, and recommendations for a self-care curriculum are made.

## Background

‘You teach us to listen to patients, to be empathic but you don’t teach us what to do about suffering’. This passing comment was thrown at me by Jane,^1^ a former student of the medical programme in which I am responsible for a formal curriculum in clinical communication skills. Jane is a woman, 12 years post-graduation. She had completed some years of postgraduate speciality training before changing direction and had been practising as a general practitioner (GP) for about 8 years. She felt burnt out by general practice and was currently taking a break and working in another area (one which could be seen as a waste of her skills).

Jane raised three points. Firstly, that though we had taught her to be empathic with her patients, she could not keep going in general practice day after day hearing all the things that she could do nothing about: the suffering due to illness but also to life in general. Secondly, that not only did we, as educators, have high expectations for patient-centred care, we also taught that preventative care must be a priority (‘the best thing that you could ever do for your patients would be to get them to stop smoking, reduce alcohol, lose weight and exercise’ she remembers being told), and all in a 10-min consultation. The bar was set too high and it was almost impossible to consistently reach it. This meant for her an almost constant feeling of inadequacy. Thirdly, important things do get missed in medical practice, almost inevitably with such time pressure. Given such high personal expectations, how does somebody already feeling as though they are falling ‘below the bar’ cope with being seen by others (patients, peers, the ‘authorities’) as having missed something important? From her perspective the shame involved in being criticized by a patient or actually making a mistake (not being perfect), even if there was no ‘harm’ to the patient, leads to doctors not being able to share and thus gain support from each other.

As a medical educator I was left with still more questions. Did our well-meaning, well-considered thought-out curriculum of person-centred, empathic medical education really contribute to this problem? Was this a small isolated experience of one very conscientious doctor, or was it more widespread? Is it a gendered phenomenon? How do these experiences contribute to stress, feelings of inadequacy, depression and burnout in the medical system? And if it is a significant problem what can we, who are educating these future doctors, do about it?

## Stress, ‘burn-out’ and mental ill-health in medical students and medical practitioners

My former student is talking about stress and burnout. Research has consistently described higher rates of mental ill-health in medical students [1] and medical practitioners [2], and rates higher still in females in both groups than in comparable populations, including higher rates of suicide [2]. In a large multicentre study, 53 % of US medical students met criteria for what is often referred to as professional ‘burnout’ (comprising measures of emotional exhaustion, depersonalization and low sense of personal accomplishment) [3]. A recent Australian survey of Australian doctors and medical students has revealed that they are more likely to experience psychological distress and suicidal thoughts and engage in risky drinking than the general community [4]. Both male and female doctors were affected, with the highest rates of severe distress being in young doctors (under 30 years) and female doctors. Furthermore, ‘burnout’ may negatively affect professional behaviour and impinge on a doctor’s ability to respond as effectively as possible to the suffering of their patients [5].

These studies raise even more questions (none of them new), and few answers. Do students enrolling in medicine have poorer mental health than other people their age, or do they have a greater vulnerability due to personality or life experiences? Are the high rates of mental ill-health in medical students produced by the experience of studying medicine? Or is it the stressful nature of the work environment as interns, trainees or more generally in medical practice? What capacities or attributes among future doctors will help them to respond to and cope with suffering? Even if there is vulnerability in the selected student populations, does the experience of medical education make it worse? If so, what should we be doing about this? How well does medical education equip doctors to respond effectively to their own health needs?

## What factors in medical education might cause or exacerbate mental ill-ease?

There are likely to be multiple factors involved including student characteristics; learning environment; exposure to patient suffering; hidden curriculum of medicine and gender [6]. Initial theories to explain the higher than expected distress in medical students centred on the difficulties experienced by students selected on the basis of high academic achievement in school, but with little understanding of the attributes necessary to treat patients in real-life conditions [2]. Whilst this is a feasible explanation, there is another possibility on the flip side.

Many medical schools now select students on desirable personal attributes for doctors [7, 8]. As a progressive medical school we led the way over 30 years ago in selecting our students by their capacity to demonstrate concern for others, and commitment to this attribute has not wavered despite increased student numbers and reviews of our selection process [8]. It is possible then that the very characteristics thought of as desirable, and selected for at admission, may also be those that make medical students more vulnerable to stress. Capacity for empathy is among the most consistently identified attributes sought among applicants to medical school and desirable competencies of medical graduates [7]. Nevertheless, student or doctor empathy is not a stable characteristic over time. It has been suggested that the formal curriculum of patient-centred communication is at odds with the hidden curriculum in the clinical environment, the place where students spend increasing amounts of time before they graduate [9, 10].

## Gender as a factor in stress, burnout and mental health problems

The proportion of female medical students has been increasing and over 50 % of medical students in many countries are now women [11]. As described above, although both male and female medical students and medical practitioners are affected it seems that females have higher rates of emotional distress than males [4] and at least one study suggests this may be related to lower levels of self-confidence in female students [12]. Females may also be expected to behave differently in clinical settings. Female GPs in the UK complained about the pressure to be more empathic, sympathetic and approachable and to take a greater interest in women’s and children’s health than their male counterparts [13], as well as looking after their colleagues and employees.

## What self-care interventions specifically related to being a doctor do pre-vocational medical programmes provide?

There are many possible points of intervention; however, we will concentrate on pre-vocational strategies. While medical education now gives importance to clinical communication skills in doctor—patient settings through teaching and assessment in the formal curriculum, this is different to an emphasis on the student’s own interpersonal (and intrapersonal) skills and personal well-being.

Few formal reports of self-care curricula for current medical students or preparation for their future roles as medical practitioners were found in the literature, and what there is has not been subject to rigorous evaluation. Enhancing self-awareness has been part of medical curricula in some universities for many years [14], but there is very little research on the effectiveness of different teaching processes. The courses are usually in the first year of medical education, as part of social and behavioural sciences when students are not in a clinical environment. As a consequence, students can be quite dismissive of this perceived ‘soft’ content.

The provision of learning about personal health, wellness and resilience is not widespread or systematic in most medical student programmes, despite being required by some accrediting bodies. The Australian Medical Council (AMC), the accrediting body for Australian and New Zealand medical schools, states: ‘The medical curriculum should specifically address issues of self-care, doctor health and the responsibility to identify and assist peers in distress’ [15]. An Australian study of a medical student well-being programme integrated into the curriculum reported significant short-term improvements in mental health and other measures [16]; another in the USA with a small number of first-year students showed positive results after a well-being intervention targeting sleep, alcohol and exercise [17]. A 10-year review of medical student health promotion programmes in the United States and Canada, however, found that the number offered had significantly decreased [18]. Recent interventions based on Mindfulness with medical students [16] and practising clinicians have shown encouraging results [19, 20] and the investigation of resilience strategies of experienced physicians provides further information to guide curriculum interventions [21]. A programme of Mindfulness Based Stress Reduction (MBSR) at Massachusetts Hospital, USA (now called ‘The Contemplative Mind in Medicine’) has been offered in their first and second year for more than 20 years [22], and outcomes from other programmes based on this model have also been positive [16].

## How can these factors be addressed in medical education?

### Managing sad and painful parts of medicine

The relief of suffering (as well as the cure of disease) must be seen as an obligation of a medical profession that is truly dedicated to the care of the sick [5]. Suffering may include physical distress but it involves the whole person ‘and has its source in challenges that threaten the intactness of the person as a complex social and psychological entity’ (p. 639). Being overwhelmed by patients’ needs and suffering are commonly mentioned by medical practitioners as barriers to exploring patients’ emotional and social issues [23]. Unexamined doctors’ emotions in response to the suffering of their patients can impact negatively on patient care and doctor well-being [23]. Medical students (and medical practitioners) need skills in monitoring distress in themselves and others, and also skills that enable them to deal with the breadth of suffering among patients.

Duckworth et al. [24] provide an outline of the developmental tasks necessary for the professional development of doctors in order to achieve equanimity in the face of patient suffering, to be helpful and effective, and to maintain healthy professional self-esteem. They include navigating a set of often conflicting tasks—caring without over-identifying, being objective rather than avoidant, and collaborating without being coercive. This can provide a framework for assisting students to identify and address core professional challenges implicit in clinical practice.

Meier et al. [23] provide a framework for clinicians to identify their emotional reactions to complex clinical situations, or circumstances in which these reactions are at risk of jeopardizing patient care.

### Developing skills in self-care as a core professional competency

A longitudinal self-care thread or theme should be incorporated as an essential part of the professional practice curriculum, no matter how ‘full’ the curriculum. It must be included in the competency framework and be assessable. The close association between the ability of doctors to care for themselves and their ability to care for their patients must be foregrounded in order for this to be seen by students as a legitimate area of study. The following four topics are recommended for inclusion in this curriculum: basic education about the medical students’ own mental health and that of their peers, helpful strategies and stigma reduction; mindfulness training which has been shown to develop resilience (noticing one’s own emotions, being able to separate emotionally from the patient’s suffering, being able to transition more effectively from work to home) and improve mental health and well-being in health professionals [16, 20]; formal and informal curricula devoted to dealing with situations when ‘things go wrong’ which would incorporate personal self-care as well as knowledge about health care institutional policies and skills in implementing procedures such as ‘Open Disclosure’ after an adverse event; a new approach to incorporating a gendered perspective on the experience of being a medical student and a medical practitioner may be needed.

## Challenges

As medical programmes increase their student numbers, distribute clinical learning to rural and distant areas, and increase the use of Web-based resources, there is a reduction in face to face hours in formal parts of the curriculum and reduced contact with medical school academic staff. Opportunities for informal interpersonal student-staff interactions with clinicians and mentors outside University settings may be theoretically available but are variable and largely unknown. The extent to which the hidden (informal) curriculum supports or contradicts the formal curriculum in the valuing of person-centred clinical communication skills and attitudes to self-care, will influence these outcomes. Of course, medical practice does not exist in a vacuum and the health care systems may continue to be depersonalizing and disempowering despite the best efforts of medical education. There is now widespread recognition of the burden on junior doctors and countries around the world are attempting to address this through different strategies including greater regulation of working hours (European Union) and in Australia, more intensive postgraduate training and support [25, 26]. The culture and arrangements for postgraduate training in different countries might merit future consideration in terms of distress caused to both men and women.

## Conclusion

High levels of psychological distress, depression and burnout in medical students and doctors have been consistently shown. Much personal suffering in these medical students and medical practitioners (and family and friends) can result. While this is obviously a complex phenomenon with no single causative factor, it behoves medical schools to ask the important questions. ‘What can be done to reduce stress, depression and anxiety, and increase the mental health and well-being of our medical students?’ ‘What can be done to increase the resilience of our medical students and to prepare them for the emotionally challenging illness care in their future professional life?’

## Footnotes

1 Jane is a pseudonym.
